# The Role of a Nurse in a Programme for Patients Undergoing Transcatheter Aortic Valve Implantation: Impact on Outcomes and Patient Experience

**DOI:** 10.3390/jcm14113944

**Published:** 2025-06-03

**Authors:** Miryam González-Cebrian, Marta Alonso-Fernández-Gatta, Ángel Víctor Hernández Martos, Sara Alonso Meléndez, Rosa Carreño Sánchez, Elena Olaya González Egido, Beatriz de Tapia Majado, Elena Calvo, Ignacio Cruz-González, Pedro L. Sánchez

**Affiliations:** 1 Cardiology Department, University Hospital of Salamanca, IBSAL, Paseo San Vicente, 58-182, 37007 Salamanca, Spain; martalonso@saludcastillayleon.es (M.A.-F.-G.); salonsome@saludcastillayleon.es (S.A.M.); rmcardeno@saludcastillayleon.es (R.C.S.); eogonzaleze@saludcastillayleon.es (E.O.G.E.); btapiam@saludcastillayleon.es (B.d.T.M.); icruz@saludcastillayleon.es (I.C.-G.); plsanchez@saludcastillayleon.es (P.L.S.); 2 CIBER-CV, Instituto de Salud Carlos III (ISCIII), 28028 Madrid, Spain; elenacb@gmail.com; 3 Cardiology Department, University Hospital of Bellvitge, 08908 Barcelona, Spain; angel.hernandez.martos@gmail.com

**Keywords:** aortic stenosis, TAVI (transcatheter aortic valve implantation), nursing, advanced practice nursing, patient experience, patient-centred care

## Abstract

**Background/Objectives:** Multidisciplinary TAVI programs are focused on improving patient-centred care. We compared outcomes in patients undergoing transcatheter aortic valve implantation (TAVI) within a multidisciplinary programme including a nurse with those of patients in the standard programme. **Methods**: This single-centre observational retrospective study includes patients with severe aortic valve disease and a TAVI indication, with the goal of comparing a nurse programme with standard practice. In the TAVI nursing programme, the nurse has several key roles: patient and family education, comprehensive assessment and procedure planification, patient and family accompaniment, complications detection during admission and follow-up, and patient experience evaluation in the post-procedure period. **Results**: 154 patients were included: 87 in the nurse programme and 67 in standard practice groups, respectively. Men comprised 52.6%, with an average age of 81 years. Both groups achieved high procedure success without differences in mortality during admission and follow-up (median 13.4 months). The nurse programme group showed better functional class more frequently and had significantly fewer emergency department visits (11.8% vs. 31.3%) and less frequency of readmission (1.2% vs. 23.4%). The TAVI nurse group reported significantly higher overall satisfaction with the process (9.8 vs. 8.9 scores), with the information received and the nurse treatment being the best rated items. **Conclusions**: A multidisciplinary programme for patients undergoing TAVI, coordinated by nurses and based on comprehensive attention that places the patients at the centre of the process, is feasible and shows high patient satisfaction.

## 1. Introduction

Since the first transcatheter aortic valve implantation (TAVI) in 2002 [1], this therapeutic option has changed the paradigm of aortic stenosis (AS) treatment, becoming the treatment of choice for patients with severe symptomatic AS and patients with high surgical risk and a valid alternative to surgical aortic valve replacement for patients with intermediate and low surgical risk [2,3,4,5,6,7]. The expansion of TAVI indications, in combination with the current ageing population, is leading to a substantial increase in the number of TAVI procedures worldwide [8,9,10].

Currently, there is a transition to minimally invasive TAVI procedures and early discharge protocols that reduce hospital stay, minimize the use of healthcare resources, and optimize final outcomes without increasing complications and mortality [11,12,13,14,15,16].

These aspects have led to the development of specific programmes for TAVI that help standardize the process, achieving excellent results and improving the efficiency of the process by placing the patient at the centre [17,18]. The implementation of multidisciplinary programmes that include nurses in a leading role, through figures such as a TAVI nurse or TAVI coordinator, has yielded positive results in planning, workflow, and maintenance of continuity of care, while also enhancing patient satisfaction and empowerment [19,20,21,22,23,24,25]. However, at present, programmes that include a TAVI nurse are anecdotal.

The aim of the study was to compare outcomes in patients undergoing TAVI within a multidisciplinary programme including a TAVI nurse with a key role in the pre-, during, and post-procedure phases with those in a conventional TAVI programme (standard clinical practice).

## 2. Materials and Methods

A single-centre retrospective study was carried out on patients with severe aortic valve disease who had an indication for intervention and who had been accepted at a multidisciplinary session for TAVI during the period from March 2022 to April 2023. We compared patients included in a TAVI nurse programme (n = 97) with those in routine clinical practice (n = 67) in the cardiology department of the University Hospital of Salamanca, a fourth-level hospital with extensive experience in structural interventionism and a national and international reference.

We selected those patients who underwent TAVI and had at least had their first outpatient follow-up visit in the TAVI nurse group.

### 2.1. Description of the TAVI Nurse Programme

The TAVI nurse programme was implemented in our hospital in March 2022. A multidisciplinary working group was created, consisting of interventional, clinical, and imaging cardiologists, coordinated by nurses familiar with the TAVI procedure from the Cardiology department (called TAVI nurses).

While the nurse in our study did not participate in the TAVI procedure itself, the TAVI nurse programme included pre-procedure, in-hospital, and post-procedure visits, giving the nurse a pivotal role in multidimensional and multidisciplinary assessment (Table 1).

#### 2.1.1. Pre-Procedural Consultation

This face-to-face session is based on three pillars: comprehensive patient assessment, patient and caregiver education, and procedure planning. The TAVI nurse conducts a multidimensional evaluation using validated questionnaires selected by the centre, assessing quality of life, frailty, cognitive function, social support, symptom burden, and functional status. The nurse also applies four centre-specific tools, designed to (1) assess patient expectations regarding the procedure, (2) measure the importance the patient places on being informed pre- and post-procedure, (3) screen for cardiac amyloidosis in selected patients, and (4) pre-identify candidates for an early discharge protocol. The aim is to optimize patient selection and procedure planning. All information collected during this consultation is systematically shared with the heart team, allowing for a more informed and personalized decision-making process regarding the indication and planning of the TAVI procedure.

The nurse also ensures that the pre-TAVI workup is complete, paying particular attention to the identification of potential risk factors or sources of complications. Education is provided to both patients and caregivers through verbal explanation, written materials, and custom-designed audiovisual resources (slides and videos). A printed leaflet is provided at the end of the visit, summarizing key instructions for the procedure day (e.g., fasting, medication management) to minimize information loss.

Additionally, patients receive a virtual tour of all units involved in their care during admission, explaining what to expect in each department.

#### 2.1.2. During Admission Care

During the hospital stay, the TAVI nurse accompanies the patient through all units involved in the procedure, including the heart day hospital, cath lab, cardiovascular intensive care, and cardiology ward. This approach promotes continuity of care and ongoing communication with both the patient and family, as well as the multidisciplinary team.

Before discharge, the nurse ensures that the patient and caregivers clearly understand the prescribed medications and post-discharge care instructions (self-care and warning signs). If a patient meets the criteria for early discharge, the nurse informs the team to initiate the centre’s fast-track discharge protocol. Evidence supports that early discharge is safe and may reduce complications, readmissions, and length of stay [21].

#### 2.1.3. Post-Discharge Follow-Up

Patients discharged early are scheduled for an in-person follow-up visit one week after the procedure to detect early complications. All patients in the programme receive an in-person nurse follow-up at one month to reassess symptoms, quality of life, and potential late complications, as well as to identify ongoing needs. Patient experience with the TAVI process is evaluated using a custom-designed questionnaire.

At six months, a telephone follow-up is conducted to monitor clinical evolution and address patient concerns. From that point onward, nurse follow-up is transitioned to primary care.

The TAVI nurse group (TN) included patients undergoing TAVI during the period from March 2022 to April 2023 who completed the TAVI nurse programme protocol visits. The nurse intervention was in addition to routine practice in the TAVI process.

### 2.2. TAVI Standard Practice Group

**Figure 1:**
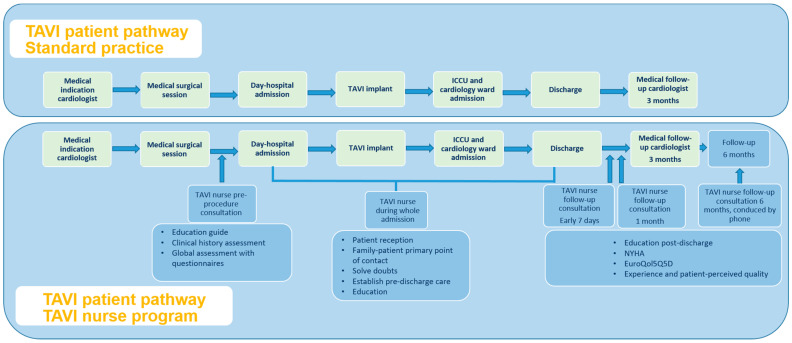
Comparison between the standard practice pathway and the nurse-led TAVI programme. Abbreviations: ICCU = intensive cardiovascular care unit, NYHA = New York Heart Association Functional Class, TAVI = transcatheter aortic valve implantation, 5Q5D = EuroQol 5 dimension.

The control group, called TAVI Standard Practice (ST), included patients undergoing TAVI during the period from March 2022 to April 2023 who were not included in the TAVI nurse programme because of different reasons (they were hospitalized at the time of the TAVI indication, required urgent implantation, or resided in other provinces managed pre-TAVI by another team). The flow of patients follows the usual practice for TAVI, consisting of the indication of valve disease intervention by the cardiologist, followed by the pre-TAVI study, acceptance to the TAVI procedure in a multidisciplinary session (heart team), admission for TAVI, and subsequent follow-up by the cardiologist in consultation three months after discharge. In this group, there was no structured involvement of a nurse in the coordination or follow-up of care (Figure 1). These patients also underwent an experience and satisfaction survey one year after the procedure.

### 2.3. TAVI Process

The pre-procedure assessment and TAVI were the same for both groups following current recommendations [10,26,27,28,29].

An angioCT of the aorta and peripheral arteries was performed in order to study the accesses (transfemoral choice), aortic annulus size for prosthesis selection, and the coronary arteries using non-invasive coronary angiography.

The elective TAVI was performed according to protocol by admission on the same day of the procedure to the Day Care Unit for preparation. TAVI is performed in the catheterization laboratory with conscious sedation in the case of transfemoral access or general anaesthesia in the case of other types of access (subclavian, transapical, or transcaval) or particular circumstances. After the procedure, the patient is admitted to the Intensive Cardiac Care Unit (ICCU) for a minimum of 24 h, followed by a stay on a hospital ward, with a planned total hospital stay of 2–4 days depending on the risk of rhythm disorders and complications, which may be shorter if the criteria established in the TAVI early-discharge protocol are met.

### 2.4. Statistical Analysis

Data collection and processing were carried out in accordance with the principles of the Declaration of Helsinki, and all data were treated anonymously. This study acquired institutional research ethics board approval from the Comité de Ética de la Investigación con Medicamentos del Área de Salud de Salamanca (number: 2021 04 761/date 25 October 2021). Prior to participation in the study, participants received a detailed explanation of the research objectives and procedures both verbally and in written form, thereby allowing them the opportunity to address any questions or concerns. Informed consent was obtained, ensuring that participants were granted sufficient time to review and sign the document. All subjects were informed of the confidentiality of their data and were assured of their right to withdraw from the study at any time without any adverse impact on their medical care.

The baseline patient characteristics, type of admission (elective or emergency), implant success, complications, length of stay, hospital survival, follow-up focused on readmissions and deaths, as well as patient satisfaction and experience of the TAVI process, were compared in both TN and ST groups.

Qualitative variables were expressed as absolute frequency (n) and percentage (%), and continuous variables as mean and standard deviation (SD) or median and interquartile range (IQR), depending on the distribution and according to normality, as evaluated by Kolmogorov–Smirnov tests. We compared the variables using a Chi-Square test or Fisher’s exact test and Student’s t-test or Mann–Whitney U test, depending on their adjustment for normality. The statistical analysis was carried out using IBM SPSS Statistics software version 22.

## 3. Results

Between March 2022 and April 2023, 166 patients were assessed for TAVI. Of these, 154 patients who met the criteria were selected as the study population.

Of 99 patients assessed in the TAVI nurse programme, we included in the NT group both those who underwent implantation and had at least the first outpatient follow-up with the nurse and those who died during admission (n = 87). The remaining patients (n = 67) made up the ST group.

In terms of the pre-procedure assessment (Table 2), the populations did not differ in baseline characteristics, with a mean age of 81 years and a high burden of cardiovascular risk factors. In both populations, the predominant valve disease was AS, with severe double lesion more frequent in the ST group. The ST group showed worse LVEF, worse functional class, and a tendency towards higher NT-proBNP levels.

With regard to the comprehensive assessment carried out in the TN group using scales, 13.6% of the patients showed a moderate to severe dependence on the Katz scale, 6% a social risk situation on the Gijon scale, 20% a suspicion of or cognitive impairment according to MMSE, a high frequency of frailty (30% frail and 47.1% pre-frail) on the FRAIL scale, and low quality of life on the EuroQol 5-dimension thermometer (median 49.83 [21.4]).

Regarding the admission during which TAVI was performed (Table 2), in most cases, the procedure was elective, and TAVI was significantly more frequent during an admission for decompensation in the ST group (43.3% vs. 8%).

Femoral access was used in virtually all groups. Implant success with respect to prosthetic function, defined as normofunction or mild aortic regurgitation (AR), was similar, although patients in the TN group showed a non-statistically significant tendency towards less residual moderate to severe aortic regurgitation (AR). The TN group had simultaneous revascularization more frequently than the ST group.

Regarding complications (Table 3), the ST group suffered significantly more strokes, cardiac tamponade, need for renal replacement therapy, and infection, as well as a non-statistically significant trend towards a higher frequency of major vascular complications (pseudoaneurysm, fistula, arterial rupture, or the need for surgical or percutaneous intervention). Pacemaker implantation, femoral haematoma, and the need for transfusion were significantly more frequent in the TN group.

The duration of hospital stay was similar in both groups (5 days). Deaths on admission were similar in both groups (3.2%), with no significant differences in terms of causes (Table 3).

Regarding outpatient follow-up (n = 149, Table 3), the TN group had an in-person follow-up in the first month in 100% of the cases, unlike the ST group. Although both groups showed improvement in functional class, the TN group more frequently showed NYHA functional class I.

The ST group had significantly more emergency department (ED) visits (mainly non-cardiac), and a higher frequency of readmissions, with the most frequent cause being infections (Table 4).

Survival at follow-up was similar in both groups, with a median of 13.4 [8.3] months.

As patients in the ST group presented a high proportion of unplanned admissions (43%) compared to the TN programme, which may introduce bias and affect the comparability of outcomes, we conducted a complementary analysis excluding those patients. The analysis continued to show results consistent with those observed in the overall population: no differences in in-hospital or follow-up mortality, and a reduction in heart failure readmissions (21.3% vs. 1.3%; *p* < 0.001) or emergency department visits (23.7% vs. 11.5%; *p* = 0.020) in the TN programme.

The experience and satisfaction questionnaire was completed by 93% in the NT group and 58% in the ST group (Table 5). Both groups reported being less symptomatic, having an improved perceived quality of life, and thinking that undergoing TAVI was worthwhile. Both groups gave high scores to the team’s care and the hospital facilities and showed high overall satisfaction, with the TN group scoring significantly better (TN group 9.8 versus ST group 8.9); the best-rated aspects were the information received and the nurse’s treatment in the programme (Table 5).

## 4. Discussion

This study compares the implementation of a TAVI programme that included a nursing role (TAVI nurse) with routine clinical practice. Our programme included a comprehensive assessment of the patient by the nurse, which was not systematically performed by the physician.

While the nurse in our study did not participate directly in the TAVI procedures, her presence in the care continuum may have indirectly influenced procedural outcomes. By ensuring comprehensive pre-procedural education, optimizing patient preparation, identifying potential risk factors early, and streamlining peri-operative coordination, the nurse likely contributed to enhanced procedural readiness and reduced complications. Post-procedurally, structured monitoring and early detection of warning signs may have facilitated timely interventions. Although our study was not designed to isolate the impact of each component, we acknowledge that the nurse’s integrative role across the care pathway could positively influence overall procedural success.

Beyond the TAVI context, the coordinating role of specialized nurses is well recognized in other cardiovascular interventions and chronic disease management. For example, in heart failure, specialist nurses not only improve treatment adherence and reduce hospitalizations but also provide continuous patient education, symptom monitoring, medication management, and psychosocial support, key factors in preventing readmissions and optimizing clinical outcomes [30,31,32]. Similarly, nurse-led protocols in atrial fibrillation ablation have demonstrated safe and efficient outpatient care with fewer readmissions [33]. These examples highlight the broader importance of nurse coordination beyond TAVI.

Including the TAVI nurse or TAVI coordinator figure in TAVI programmes is widely supported internationally. The USA, UK, and Canada [20] were pioneers in its development with early adoption, and in recent years, there has been growing interest in Europe and Asia, although it has been integrated in different ways and with responsibilities varying from centre to centre. The implementation of TAVI coordinators has been spreading with excellent results [21,22], and in many centres, it is a role assumed by nurses, as in our study.

### 4.1. TAVI Nurses in Health Education

Nursing health education and information to the patient and relatives were some of the main objectives of our TN programme, helping the individual become an informed patient [22]; this was reflected in the process evaluation surveys, where information and treatment by the nurse were the most highly rated points. Shared decision-making—ensuring that the patients understand their disease, the TAVI procedure itself, and the limitations and risks—was a pillar of our project. In addition, at discharge, the patient was educated about warning signs, medication, progressive activity, and how to seek help if needed [17,20,34].

### 4.2. Expectation Management by TAVI Nurse

Pre-procedure beliefs, expectations, and anxiety have a great impact on recovery and on the quality of life perceived by patients [17,18,20,21,22,34]. In our study, in the pre-procedure visit, the TAVI nurse managed the patient’s motivations and expectations for the TAVI using self-developed material. The aim was to avoid unfounded expectations, help the patient’s conception of the procedure to be as realistic as possible [17,18,20,21,22,34], and to support the patient’s decisions. Patients’ expectations of the procedure are collected and then compared at follow-up to determine whether the results were in line with the expectations generated, which was the case in 87% of cases in the TN group.

A study in patients undergoing cardiac surgery for aortic valve replacement [35] showed that a brief pre-surgery psychological intervention aimed at setting realistic expectations improved post-operative outcomes, shortened length of stay, and facilitated both a quicker return to daily life and a higher perceived quality of life. Nursing education in our TN programme was likely to help modulate appropriate and realistic expectations in each case.

### 4.3. Comprehensive Assessment

Another novelty of our programme was to include a multidimensional, comprehensive pre-procedure assessment of the patient by a multidisciplinary team, including nursing assessment. Frailty, cognitive impairment, dependency, and social risk situations are frequent conditions in elderly patients undergoing TAVI, which may negatively affect mortality and morbidity, lead to a poorer quality of life after a TAVI procedure, and lead to longer hospital stays and subsequent readmissions [18,20,21,36,37,38,39]. The advanced age and characteristics of patients undergoing TAVI justify their inclusion in the comprehensive assessment to assist in appropriate patient selection and predict which patients would benefit most and which would not, allowing modulation of the indication for TAVI. The experience of nurses in these areas could significantly strengthen a holistic approach. In our programme, the nurse performed the non-cardiologic multidimensional assessment through validated questionnaires, which is a competence increasingly assumed by nurses and common in centres with TAVI programmes [17,18,21,22]. The TN group detected a high frequency of frailty and low quality of life, and a significant percentage of patients with suspected or impaired cognitive impairment, social risk, and moderate dependency. Detection of these conditions could lead to improvements in these dimensions through preparation and appropriate treatment. In the ST group, this multidimensional assessment was not systematically performed, so the TAVI nurse could be instrumental in facilitating the regulated assessment of relevant comorbidities.

### 4.4. Communication with the Team and Planning of the Procedure and Discharge

In our programme, the TAVI nurse coordinated the process through regular meetings and communication with the multidisciplinary team to discuss the development and evolution of the patient in each phase [17,18], individualizing planning of care oriented to early discharge, reinforcement of the patients and family accompaniment, and facilitating transition to home and follow-up. In our TN programme, the nurse was the point of contact for the patient and family throughout the process and could also be the link with the medical team. The COORDINATE [22] study concludes that a programme with a TAVI coordinator could decrease the time between the diagnosis of AS and the TAVI procedure, reduce the working hours of the team staff, and support the programme team and patients during admission.

### 4.5. TAVI Nurse Impact on TAVI Outcomes (Results, Complications, Survival, and Discharge)

In terms of the procedure’s success, the TN group showed a non-statistically significant tendency towards a higher percentage of normofunctioning prosthesis or with mild AR compared to the ST group. Differences in the aortic valve disease characteristics of the groups (a higher percentage of severe double lesions in the ST group) may have influenced the outcome.

Regarding in-hospital complications, we observed a higher frequency of stroke, cardiac tamponade, infection, major vascular complications, and vascular intervention in the ST group. Vascular complications are significantly associated with a higher rate of post-interventional morbidity and mortality [40], making it important to discuss planning and prevention, which nurses can help with. On the other hand, the TN group showed higher rates of pacemaker implantation, femoral haematoma, and the need for transfusion.

Systematic intervention in the pre-procedural assessment by nurses, in addition to medical action, could help to identify possible sources of complications. Patient and family education and follow-up could facilitate early detection of complications on admission and post-discharge. However, the incidence of adverse events is different from other studies, such as the COORDINATE study [22], despite similar baseline patient characteristics in terms of age, gender, morbidity, cardiovascular risk factors, and the presence of a TAVI coordinator. Therefore, we cannot attribute the differences in complication rates between the two groups to the TAVI nurse intervention, a point that would require further study.

In-hospital mortality was similar in both groups in our population, coinciding with other programmes that incorporated a TAVI coordinator [22]. In this sense, nursing intervention in the TAVI process seems to improve aspects related to preparation, education, and monitoring of complications, but would not influence peri-procedural mortality.

Regarding hospital stay, although we did not find statistically significant differences, the TN group had a shorter crude hospital stay (4 (4) vs. 5 (6) days, *p* = 0.243), data that coincide with the literature [18,19,25].

The reduction in hospital stay in TAVI nurse programmes may be due to improved procedure planning and organized early discharge. The TAVI nurse helped to improve patient information and preparation for the procedure, while preparing for an adequate discharge and maintaining continuity of care, as in other similar programmes [21,22]. However, the ST group was admitted for decompensation at the time of TAVI implantation in a higher percentage of cases, which could affect the length of stay in this group.

The implementation of early discharge protocols, as well as a minimalist TAVI approach, helps to reduce length of stay, readmissions, complications, and the use of resources without affecting patient safety [11,12,13,14,15,16,20,21,34]. Based on this evidence, together with our minimally invasive approach to the procedure (preferential femoral access and conscious sedation), we have incorporated an early discharge protocol (24 h after implantation) in our TN programme since 2023, summarized in appropriate candidate pre-selection, compliance with safety criteria during admission, and adequate post-discharge planning [20,22]. To this end, we developed a checklist that the nurse uses during the pre-TAVI consultation to guide the pre-selection of candidate patients, which is subsequently carried out if all the conditions are met.

The BENCHMARK study [23] raises the need to unify criteria for TAVI procedures since the quality of care is highly variable across Europe. It establishes the TAVI coordinator or TAVI nurse as an element to ensure a correct flow of care and continuity of care. It states that TAVI processes should be increasingly streamlined to reduce resources, optimize patient flow, and above all, improve quality of life and patient experience [23]. Moreover, the study by Saia et al. demonstrates that incorporating these coordination roles improves discharge management and increases patient satisfaction without compromising safety [25].

### 4.6. TAVI Nurse in Follow-Up

The face-to-face follow-up in the TN group was 100%, significantly higher than in the ST group. The systematization of follow-up by the nurse, in addition to the usual medical appointment, could have influenced the follow-up percentage in the TN group.

Although survival during follow-up was similar for both groups, the TN group patients were more often in the I NYHA functional class and had significantly fewer ED visits and readmissions.

Early, systematized, face-to-face follow-up, with an available point of contact (TAVI nurse), could allow the patient to consult and anticipate visits in cases of detecting possible complications or decompensation. On the other hand, the additional education and information provided by the nurse in the pre-procedural visits and during admission could influence the ability of TN patients and relatives to detect complications and consult appropriately, allowing them to differentiate between these and situations of normal post-procedural recovery. Again, nursing intervention would not change the prognosis in terms of mortality. However, we believe that follow-up with the TAVI nurse throughout the process could have helped in the early detection of complications, as well as in avoiding unnecessary visits to the ED and reducing readmissions.

On the other hand, in the ST group, TAVI was more frequently performed in the context of decompensation during an emergency hospital admission, which, together with the differences in patient characteristics between the two study groups, may have influenced the higher rate of subsequent ED visits and readmissions.

### 4.7. TAVI Nurse’s Impact on Patient Satisfaction with the Process

The implementation of a TAVI nurse’s role allowed us to improve overall patient satisfaction with the programme. In our study, although both groups showed high satisfaction with the process, the TN patients gave a significantly higher score (9.8 vs. 8.9 out of 10) to the programme, with the information received and treatment by the nurse being the highest-rated items, coinciding with other studies [20,22]. In our opinion, the nursing intervention facilitated patient-centred care. The accompaniment of the patient and family by the TAVI nurse, integrating nursing care, could be one of the causes that helps to improve patient experience and satisfaction. However, some differences in the baseline characteristics of both populations and the higher percentage of TAVI procedures occurring during decompensation in the ST group could also influence their lower satisfaction.

Therefore, it is necessary to measure for improvement and include the patient perspective to ensure that processes are patient-centred and tailored to patients’ needs. Registries of frailty, mortality, complications, length of stay, clinical outcomes, and cost-effectiveness should be established to evaluate TAVI programmes and monitor their growth [17,18,21]. Evaluating care processes, as in our case, helps to improve outcomes and resource management [17,18].

### 4.8. Limitations

This was a single-centre study, which may limit the generalizability of the results. The analysis was retrospective, with the inherent methodological limitations of this design. The groups were not randomized, and differences in baseline characteristics may have influenced the outcomes, warranting further investigation. However, contemporary cohorts were selected to minimize variability in the TAVI procedure and clinical practice over time. To our knowledge, this is the first study to compare outcomes between patients undergoing TAVI within a multidisciplinary programme that includes a dedicated TAVI nurse and those receiving standard care.

The observed improvements in patient adherence and satisfaction may also have been influenced by the Hawthorne effect. Patients receiving structured follow-up and increased attention from the nursing team may have altered their behaviour, leading to improved adherence and perceived satisfaction, irrespective of the actual clinical intervention. This potential bias should be considered when interpreting subjective outcome measures.

Furthermore, implementing a nurse-coordinated care programme requires a well-structured clinical setting with trained staff and institutional support. Reproducing this model in other healthcare systems may present organizational challenges, especially in resource-limited settings or where multidisciplinary collaboration is less established. The success of such programmes largely depends on the availability of institutional resources and coordinated team-based care.

Additionally, although this study provides valuable insights into the impact of a nurse-led care model, further prospective, randomized, multicentre studies are needed to confirm the generalizability of these findings and to specifically assess the impact of nurse-coordinated TAVI programmes on in-hospital and post-discharge mortality, as well as other clinical outcomes. This study did not include a cost-effectiveness analysis, which is also relevant when considering the broader implementation of such models.

The causes of complications were not explored in depth, which may limit the interpretation of safety-related outcomes. Moreover, the median follow-up of 13.4 months may restrict the evaluation of long-term results. These aspects warrant further investigation in future studies.

## 5. Conclusions

A programme for patients undergoing TAVI based on comprehensive and multidisciplinary care, which includes coordination by specialized nurses and places the patient at the centre of the process, ensuring quality of care, clinical management efficiency, and patient safety, is feasible. The TAVI nurse helps reinforce patient education, information, and communication with the team, facilitates a comprehensive and multidimensional assessment, and provides support throughout the entire process, contributing to maintaining quality and continuity of care as well as improving the patient’s experience, without significantly influencing mortality. It is important to highlight that pre-TAVI decisions are complex and require thorough evaluation by the heart team. In this context, the TAVI nurse emerges as a valuable figure to implement in multidisciplinary care programs.

## Figures and Tables

**Table 1:** Nurse role protocol during each phase in the TAVI nurse programme.

TAVI Nurse Programme		TAVI Nurse Role Pre-Procedure		TAVI Nurse Role During Admission	TAVI Nurse Role Post-Procedure
NURSE VISITS		Duration 45 min 7–15 days prior to the TAVI procedure		Reception of patients and visits to relatives in the different units: Day Care Hospital Catheterization laboratory Cardiovascular ICCU Hospitalization	Duration 20–30 min Selected patients: Early post-discharge (7 days) One month post-discharge 6 months (by phone)
PATIENT AND FAMILY EDUCATION		▪ Health education through slide shows ▪ Paper brochure		Discharge preparation: Self-care Medication Warning signs Resolving doubts	Follow-up care Needs Self-care Medication Warning signs Resolving doubts Transition to primary care team
COMMUNICATION WITH THE TAVI TEAM		X		X	X
TAVI PROCEDURE ASSESSMENT	Assessment of MEDICAL HISTORY	X		X	X
	Pre-selection of candidates for the EARLY DISCHARGE PROTOCOL *.	X			
COMPREHENSIVE ASSESSMENT	Symptomatology: NYHA		X		X
	Dependency: KATZ SCALE		X		
	Social and family support: GIJON		X		
	Cognitive impairment: MMSE		X		
	Fragility: FRAGILE		X		X
	Quality of life: 5Q5D		X		
	Screening: CARDIAC AMYLOIDOSIS suspicion		X		
	INFORMATION AND EXPECTATIONS		X		
FOLLOW-UP ASSESSMENT	Detection of COMPLICATIONS			X	X
	Perceived QUALITY OF LIFE AND EXPERIENCE				X

* Included in the second phase of the implementation of the TAVI nurse programme in April 2023. Abbreviations: ICCU = intensive cardiovascular care unit, MMSE = mini-mental state examination, NYHA = New York Heart Association Functional Class, TAVI = transcatheter aortic valve implantation, 5Q5D = EuroQol 5 dimension.

**Table 2:** Pre-TAVI assessment.

Pre-Procedural Assessment TN vs. ST					
			Programme		*p* Value
		All (n = 154)	TN (n = 87)	ST (n = 67)	
Baseline characteristics	Age (mean + SD)	81.6 ± 7	81.8 ± 6	81.2 ± 8	0.607
	Sex male (n, %)	81 (52.6%)	46 (52.9%)	35 (52.2%)	1
	Arterial hypertension (n, %)	122 (79.2%)	69 (79.3%)	53 (79.1%)	1
	Diabetes mellitus (n, %)	42 (27.3%)	21 (24.1%)	21 (31.3%)	0.364
	Dyslipidaemia (n, %)	91 (59.1%)	55 (63.2%)	36 (53.7%)	0.251
	Peripheral arterial disease (n, %)	17 (11%)	10 (11.5%)	7 (10.4%)	1
	CKD (n, %)	44 (28.6%)	21 (24.1%)	23 (34.3%)	0.152
Valvulopathy characteristics	Aortic valve disease (n, %)				0.029
	Stenosis	117 (76%)	68 (78.2%)	49 (73.1%)	
	Regurgitation	16 (10.4%)	12 (13.8%)	4 (6%)	
	Both (≥moderate)	21 (13.6%)	7 (8%)	14 (20.9%)	
	LVEF (%) (mean + SD)	55.7 ± 13	59 ± 12	51.7 ± 14	0.001
	RV dysfunction (n, %)	15 (9.7%)	8 (9.2%)	7 (10.4%)	0.721
	NYHA				0.037
	I	10 (6.6%)	9 (10.6%)	1 (1.5%)	
	II	74 (48.7%)	42 (49.4%)	32 (47.8%)	
	III	51 (33.6%)	27 (21.8%)	24 (35.8%)	
	IV	17 (11.2%)	7 (8.2%)	10 (14.9%)	
Analytics	Creatinine (median, IQR)	1 [0.45]	1 [0.5]	1 [0.45]	0.922
	NT-proBNP (median, IQR)	2484 [5154]	1933 [3225]	4042 [10,152]	0.089
	Haemoglobin (mean + SD)	13 ± 2	13.2 ± 2	12.9 ± 2	0.438

Abbreviations: CKD = chronic kidney disease, IQR = interquartile range, LVEF = left ventricular ejection fraction, NT-proBNP = N-terminal natriuretic brain peptide, NYHA = New York Heart Association Functional Class, RV = right ventricular, SD = standard deviation, ST = standard TAVI, TAVI = transcatheter aortic valve implantation, TN = TAVI nurse.

**Table 3:** TAVI procedure admission.

TAVI Procedure Admission TN vs. ST				
		Programme		*p* Value
	All (n = 154)	TN (n = 87)	ST (n = 67)	
TAVI success Normofunctioning Mild AR Moderate-severe AR Periprocedure ETI Access Transfemoral Subclavian Transcaval TAVI during urgent unplanned admission Simultaneous coronary revascularisation	82 (53.2%) 61 (39.6%) 11 (7.1%) 5 (3.2%) 151 (98.1%) 2 (1.3%) 1 (0.6%) 36 (23.4%) 24 (15.6%)	42 (48.3%) 41 (47.1%) 4 (4.6%) 2 (2.3%) 85 (97%) 1 (1.1%) 1 (1.1%) 7 (8%) 10 (14.9%)	40 (59.7%) 20 (29.9%) 7 (10.4%) 3 (4.5%) 66 (98.5%) 1 (1.5%) 0 (0%) 29 (43.3%) 14 (16.1%)	0.059 0.653 0.321 <0.001 0.027
Complications (n, %) Pacemaker Ictus Vascular Haematoma Mayor Unplanned surgical intervention Unplanned endovascular stenting Thrombin embolization Transfusion Cardiac tamponade Renal replacement therapy Infection	37 (24%) 7 (4.5%) 33 (21.4%) 34 (22.1%) 4 (2.6%) 17 (11%) 8 (5.2%) 13 (8.4%) 5 (3.2%) 2 (1.3%) 18 (11.75)	25 (28.7%) 3 (3.4%) 24 (27.6%) 18 (20.7%) 2 (2.3%) 8 (11.9%) 7 (10.4%) 9 (10.3%) 2 (2.3%) 0 (0%) 4 (4.6%)	12 (17.9%) 4 (6%) 8 (11.9%) 16 (23.9%) 2 (3%) 9 (10.3%) 1 (1.1%) 4 (6%) 3 (4.5%) 2 (3%) 14 (20.9%)	0.018 0.024 0.002 0.052 0.053 0.096 0.000 0.030 0.040 0.011 0.002
Admission duration (days) (median, IQR)	5 [4]	4 [4]	5 [6]	0.243
Exitus during admission (n, %) Cause of death on admission (n, %) Cardiac Procedure complication Infection Neurological	5 (3.2%) 2 (1.3%) 1 (0.6%) 1 (0.6%) 1 (0.6%)	2 (2.3%) 2 (2.3%) 0 (0%) 0 (0%) 0 (0%)	3 (4.5%) 0 (0%) 1 (1.5%) 1 (1.5%) 1 (1.5%)	0.653 0.121

Abbreviations: AR = aortic regurgitation, ETI = endotracheal intubation, IQR = interquartile range, SD = standard deviation, ST = standard TAVI, TAVI = transcatheter aortic valve implantation, TN = TAVI nurse.

**Table 4:** Follow-up after TAVI.

Follow-Up TN vs. ST				
		Programme		*p* Value
	All (n = 149)	TN (n = 85)	ST (n = 64)	
Face-to-face 1 month, clinic (n, %)	135 (90.6%)	85 (100%)	50 (78.1%)	0.001
Follow-up time (months) (median, IQR)	13.4 [8.3]	12.1 [9]	14.8 [8.1]	0.079
NYHA follow-up (n, %) Unknown I II III IV	8 (5.4%) 80 (53.7%) 37 (24.8%) 4 (2.7%) 0 (0%)	6 (7.1%) 57 (67.1%) 13 (15.3%) 3 (3.5%) 0 (0%)	15 (23.4%) 23 (35.9%) 24 (37.5%) 1 (1.6%) 0 (0%)	<0.001
Readmission (n, %) Cause of readmission (n, %) Cardiac TAVI complication Stroke Vascular Infection Other ED visit (n, %) Cause of ED visit (n, %) Cardiac TAVI complication Non-cardiac	16 (10.7%) 1 (0.7%) 2 (1.3%) 1 (0.7%) 1 (0.7%) 6 (4%) 2 (1.3%) 30 (20.1%) 8 (5.4%) 1 (0.7%) 19 (12.8%)	1 (1.2%) 0 (0%) 0 (0%) 0 (0%) 0 (0%) 1 (1.2%) 0 (0%) 10 (11.8%) 2 (2.4%) 0 (0%) 8 (9.4%)	15 (23.4%) 1 (1.6%) 2 (3.1%) 1 (1.6%) 1 (1.6%) 6 (9.4%) 2 (3.1%) 20 (31.3%) 6 (9.4%) 1 (1.6%) 11 (17.2%)	<0.001 0.010 <0.001 0.054
Exitus in follow-up (n, %) Cause of death in follow-up (n, %) Cardiac Neurological Infectious Other Unknown	18 (12.1%) 5 (3.4%) 2 (1.3%) 5 (3.4%) 5 (3.4%) 1 (0.7%)	13 (15.3%) 3 (3.5%) 1 (1.2%) 2 (2.4%) 5 (5.9%) 1 (1.2%)	5 (7.8%) 2 (3.1%) 1 (1.6%) 3 (4.7%) 0 (0%) 0 (0%)	0.183 0.182

Abbreviations: ED = emergency department, IQR = interquartile range, NYHA = New York Heart Association Functional Class, ST = TAVI standard TAVI, TAVI = transcatheter aortic valve implantation, TN = TAVI nurse.

**Table 5:** Experience and satisfaction questionnaire after TAVI.

Experience and Satisfaction After TAVI TN vs. ST				
		Programme		*p* Value
Questionnaire Completed (n, %)	All (n = 116) (77.9% of Survivors)	TN (n = 79) (92.9% of Survivors)	ST (n = 37) (57.8% of Survivors)	
Perceived quality of life - Expectations: having been able to return to the activities you expected - Perceived symptomatology compared to pre-TAVI - Worse - The same - Better - Much better - Perceived health in comparison to pre-TAVI - Good/very good - Fair/bad - Being worthwhile to undergo the procedure	99 (85.3%) 4 (3.4%) 10 (8.6%) 50 (43.1%) 47 (40.5%) 99 (85.3%) 12 (10.3%) 105 (90.5%)	69 (87.3%) 2 (2.5%) 9 (11.4%) 28 (35.4%) 36 (45.6%) 65 (82.3%) 10 (12.7%) 71 (89.9%)	30 (81.1%) 2 (5.4%) 1 (2.7%) 22(59.5%) 11 (29.7%) 34 (92%) 2 (5.4%) 34 (92%)	0.340 0.076 0.125 0.178
Experience and satisfaction process - Recommend TAVI to patients in the same situation - Adequate information received - Attention received - Very good - Good - Satisfaction with TAVI programme (score 0–10) (mean + SD) - Correct decision on TAVI - Facilities (comfort, cleanliness, tidiness) - Regular - Good - Very good - Treatment, time spent with nursing care - Regular - Good - Very good - Information received enabled them to arrive at the procedure more prepared and at ease.	107 (92.2%) 108 (93.1%) 89 (76.7%) 23 (19.8%) 9.5 ± 0.9 104 (89.7%) 4 (3.4%) 40 (34.5%) 68 (58.6%) 3 (2.6%) 18 (15.5%) 91 (78.4%) 107 (92.2%)	72 (91.1%) 75 (94.9%) 62 (78.5%) 13 (16.5%) 9.8 ± 0.5 71 (89.9%) 3 (3.8%) 25 (31.6%) 47 (59.5%) 1 (1.3%) 6 (33.3%) 68 (86.1%) 74 (93.7%)	35 (94.6%) 33 (89.2%) 27 (73%) 10 (27%) 8.9 ± 1.3 33 (89.2%) 1 (2.7%) 15 (40.5%) 21 (56.8%) 2 (5.4%) 12 (32.4%) 23 (62.2%) 33 (89.2%)	0.685 0.026 0.104 <0.001 0.696 0.289 0.001 0.454

Abbreviations: SD = standard deviation, ST = standard TAVI, TAVI = transcatheter aortic valve implantation, TN = TAVI nurse.

## Data Availability

Data are available upon request from the authors. The raw data supporting the conclusions of this article will be made available by the authors on request.

## Abbreviations

AS	aortic stenosis
TAVI	transcatheter aortic valve implantation
TN	TAVI nurse
ICU	intensive care unit
MMSE	mini-mental state examination
NYHA	New York Heart Association Functional Class
5Q5D	EuroQol 5 dimension
CKD	chronic kidney disease
IQR	interquartile range
LVEF	left ventricular ejection fraction
NT-proBNP	N-terminal natriuretic brain peptide
NYHA	New York Heart Association Functional Class
RV	right ventricular
SD	standard deviation
AR	aortic regurgitation
ST	standard TAVI
ED	emergency department
